# Clinical Improvisatory Music for Alzheimer's Disease Anxiety and Caregivers (CIMAC): Preliminary Behavioral and Network Modulation Findings from Ongoing Trials

**DOI:** 10.1002/alz70858_104068

**Published:** 2025-12-26

**Authors:** Kate Vidano, Jordan Quinn Behn, Clara Takarabe, Borna Bonakdarpour

**Affiliations:** ^1^ Northwestern Music and Medicine Program, Chicago, IL, USA; ^2^ Northwestern University, Evanston, IL, USA; ^3^ Mesulam Center for Cognitive Neurology and Alzheimer's Disease, Chicago, IL, USA; ^4^ Northwestern University Feinberg School of Medicine, Chicago, IL, USA

## Abstract

**Background:**

Approximately 40% of individuals with Alzheimer's disease (AD) experience anxiety (AD‐A), decreasing their quality of life and burdening caregivers. Anxiety medications can have negative side effects, prompting exploration of non‐pharmacological interventions like music. Here we present preliminary data for ongoing trials exploring the feasibility of using Clinically Designed Improvisatory Music (CDIM), previously shown effective in decreasing anxiety in healthy individuals, in reducing anxiety in AD‐A and caregiver burden, along with its effects on four relevant brain networks.

**Method:**

Nine individuals with AD‐A and 10 caregivers have completed the study so far. Using delayed enrollment, participants underwent two pre‐CDIM and one post‐CDIM evaluation. CDIM included 8 sessions, each 30 minutes, featuring slow, melodic lines in multiple 2‐minute interspaced segments played by a certified clinical music practitioner. Anxiety (RAID), caregiver burden (ZBI), and blood pressure (BP) heart rate (HR, and respiratory rate (RR) were recorded. Changes across evaluations were analyzed with the Mann‐Whitney U test. Resting functional magnetic resonance imaging (fMRI) resting connectivity was compared across four networks: Default Mode (DMN), Salience (SN), Reward (RN), and Auditory Limbic (ALN).

**Results:**

Preliminary results showed a decrease in RAID (*p* = 0.04) and ZBI scores (*p* = 0.01) following CDIM compared to no intervention. Caregivers had reduced systolic BP (*p* = 0.01), diastolic BP (*p* = 0.04), and RR (*p=0.002*). fMRI results for AD‐A participants revealed increased connectivity in SN, ALN, RN, and decreased connectivity in anterior DMN. Caregivers showed increased connectivity in posterior DMN, SN, and ALN. In AD‐A, the correlation between RN, and decreased RAID showed a trend towards significance (*p* = 0.08). Increased DMN and ALN connectivity for caregivers negatively correlated with ZBI scores (*p* < 0.05).

**Conclusion:**

Our findings so far indicate that CDIM reduces anxiety and caregiver burden. Caregivers also had decreased BP, and RR. Findings suggest 8 sessions of CDIM are feasible to induce symptomatic, physiologic, and neural effects. We believe CDIM transitions the autonomic and emotional networks to a calmer state through entrainment. Increased ALN, RN, and DMN connectivity suggests CDIM may help modulate abnormal network connectivity, reducing anxiety symptoms. Recruitment and fMRI data collection are ongoing to expand these results.

